# *catena*-Poly[[tris­(4-fluoro­benz­yl)tin(IV)]{μ-[(*N*,*N*-diisopropylcar­bamo­thioyl)sulfanyl]acetato-κ^2^ *O*:*O*′}]

**DOI:** 10.1107/S1600536810005933

**Published:** 2010-02-20

**Authors:** Thy Chun Keng, Kong Mun Lo, Seik Weng Ng

**Affiliations:** a Department of Chemistry, University of Malaya, 50603 Kuala Lumpur, Malaysia

## Abstract

In the title compound, [Sn(C_7_H_6_F)_3_(C_9_H_16_NO_2_S_2_)]_*n*_, the Sn atom is coordinated in a slightly distorted, *trans*-C_3_SnO_2_ trigonal-bipyramidal environment. Symmetry-related Sn atoms are bridged by diisopropyl­dithio­carbamoylacetato ligands, forming a one-dimensional polymer along [001].

## Related literature

Trialkyl­tin carboxyl­ates are generally carboxyl­ate-bridged polymers; see: Ng *et al.* (1988 ▶). For the direct synthesis of substituted tribenzyl­tin chlorides, see: Sisido *et al.* (1961 ▶). For the synthesis of dithio­carbamoylacetic acids, see: Nachmias (1952 ▶). For background to the triorganotin derivatives of dithio­carbamylacetic acids, see: Ng & Kumar Das (1991 ▶).
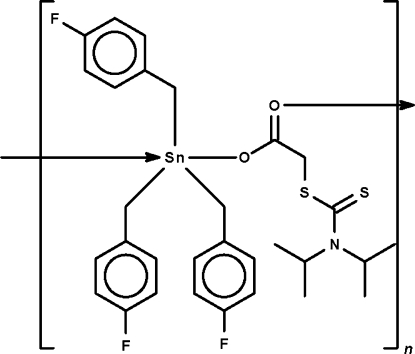


## Experimental

### 

#### Crystal data

[Sn(C_7_H_6_F)_3_(C_9_H_16_NO_2_S_2_)]
*M* *_r_* = 680.39
Monoclinic, 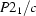

*a* = 11.2496 (2) Å
*b* = 25.7598 (5) Å
*c* = 11.4216 (2) Å
β = 105.427 (1)°
*V* = 3190.58 (10) Å^3^
*Z* = 4
Mo *K*α radiation
μ = 0.98 mm^−1^
*T* = 293 K
0.30 × 0.20 × 0.10 mm

#### Data collection

Bruker SMART APEX diffractometer
Absorption correction: multi-scan (*SADABS*; Sheldrick, 1996 ▶) *T*_min_ = 0.759, *T*_max_ = 0.909
21159 measured reflections
7090 independent reflections
5217 reflections with *I* > 2σ(*I*)
*R*_int_ = 0.050

#### Refinement

*R*[*F* ^2^ > 2σ(*F* ^2^)] = 0.054
*wR*(*F* ^2^) = 0.159
*S* = 1.09
7090 reflections
352 parameters
H-atom parameters constrained
Δρ_max_ = 1.27 e Å^−3^
Δρ_min_ = −1.18 e Å^−3^

### 

Data collection: *APEX2* (Bruker, 2009 ▶); cell refinement: *SAINT* (Bruker, 2009 ▶); data reduction: *SAINT*; program(s) used to solve structure: *SHELXS97* (Sheldrick, 2008 ▶); program(s) used to refine structure: *SHELXL97* (Sheldrick, 2008 ▶); molecular graphics: *X-SEED* (Barbour, 2001 ▶); software used to prepare material for publication: *publCIF* (Westrip, 2010 ▶).

## Supplementary Material

Crystal structure: contains datablocks global, I. DOI: 10.1107/S1600536810005933/lh2996sup1.cif

Structure factors: contains datablocks I. DOI: 10.1107/S1600536810005933/lh2996Isup2.hkl

Additional supplementary materials:  crystallographic information; 3D view; checkCIF report

## Figures and Tables

**Fig. 1.**
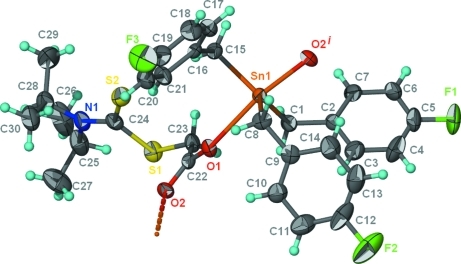
Thermal ellipsoid plot (Barbour, 2001) of a portion of polymeric Sn(C7H6F)3(C9H16N2O2S2) at the 50% probability level. Hydrogen atoms are drawn as spheres of arbitrary radius [symmetry code: (i) x, -y+1/2, z+1/2].

## supplementary crystallographic information

### Comment

Part of the one-dimensional polymer of the title compound is shown in Fig. 1.

### Experimental

Diisopropyldithiocarbomylacetic acid was synthesized from diisopropylamine, carbon disulfide and chloroacetic acid (Nachmias, 1952). Tri(*p*-fluorobenzyl)tin chloride was prepared by direct synthesis from *p*-fluorobenzyl chloride and tin powder in a mixture of toluene and water (Sisido *et al.*, 1961). The triorganotin chloride was hydrolyzed with dilute sodium hydroxide solution to give tri(*p*-fluorobenzyl)tin hydroxide. The carboxylic acid (0.10 g, 0.43 mmol) and the organotin hydroxide (0.20 g, 0.43 mmol) were heated in ethanol (100 ml) for 1 hour. After filtering the mixture, colorless crystals were obtained upon slow evaporation of the filtrate.

### Refinement

Hydrogen atoms were placed in calculated positions (C–H 0.93–0.97 Å) and were treated as riding on their parent atoms, with *U*(H) set to 1.2–1.5 times *U*_eq_(C). The final difference Fourier map had a peak/hole in the vicinity of Sn1. The magnitudes decreased when the 2θ limit was lowered to 50 °. The Sn<-O bridging bond is somewhat long [Sn1-O1 2.500 (4)Å] and may lead to inefficient packing and hence the reason for the larger than normal voids in the structure [ca. 142 Å^3^].

### Crystal data

**Table:**

[Sn(C_7_H_6_F)_3_(C_9_H_16_NO_2_S_2_)]	*F*(000) = 1384
*M**_r_* = 680.39	*D*_x_ = 1.416 Mg m^−^^3^
Monoclinic, *P*2_1_/*c*	Mo *K*α radiation, λ = 0.71073 Å
Hall symbol: -P 2ybc	Cell parameters from 5664 reflections
*a* = 11.2496 (2) Å	θ = 2.4–28.3°
*b* = 25.7598 (5) Å	µ = 0.98 mm^−^^1^
*c* = 11.4216 (2) Å	*T* = 293 K
β = 105.427 (1)°	Block, colorless
*V* = 3190.58 (10) Å^3^	0.30 × 0.20 × 0.10 mm
*Z* = 4	

### Data collection

**Table:**

Bruker SMART APEX diffractometer	7090 independent reflections
Radiation source: fine-focus sealed tube	5217 reflections with *I* > 2σ(*I*)
graphite	*R*_int_ = 0.050
ω scans	θ_max_ = 27.5°, θ_min_ = 1.6°
Absorption correction: multi-scan (*SADABS*; Sheldrick, 1996)	*h* = −14→14
*T*_min_ = 0.759, *T*_max_ = 0.909	*k* = −31→33
21159 measured reflections	*l* = −14→14

### Refinement

**Table:**

Refinement on *F*^2^	Primary atom site location: structure-invariant direct methods
Least-squares matrix: full	Secondary atom site location: difference Fourier map
*R*[*F*^2^ > 2σ(*F*^2^)] = 0.054	Hydrogen site location: inferred from neighbouring sites
*wR*(*F*^2^) = 0.159	H-atom parameters constrained
*S* = 1.09	*w* = 1/[σ^2^(*F*_o_^2^) + (0.0769*P*)^2^ + 3.6716*P*] where *P* = (*F*_o_^2^ + 2*F*_c_^2^)/3
7090 reflections	(Δ/σ)_max_ = 0.001
352 parameters	Δρ_max_ = 1.26 e Å^−^^3^
0 restraints	Δρ_min_ = −1.18 e Å^−^^3^

### Fractional atomic coordinates and isotropic or equivalent isotropic displacement parameters (Å^2^)

**Table:**

	*x*	*y*	*z*	*U*_iso_*/*U*_eq_	
Sn1	0.66161 (3)	0.225810 (13)	0.57255 (3)	0.03172 (13)	
S1	0.70901 (16)	0.42111 (6)	0.37053 (14)	0.0515 (4)	
S2	0.45463 (17)	0.38461 (7)	0.35217 (17)	0.0594 (4)	
F1	1.1949 (5)	0.1403 (3)	0.9730 (5)	0.131 (2)	
F2	1.0347 (5)	0.05335 (19)	0.3995 (7)	0.123 (2)	
F3	0.1222 (4)	0.1519 (2)	0.2507 (4)	0.0944 (15)	
O1	0.6613 (4)	0.26909 (14)	0.3765 (3)	0.0399 (9)	
O2	0.6790 (3)	0.32296 (14)	0.2303 (3)	0.0395 (8)	
N1	0.5111 (5)	0.47966 (18)	0.2933 (4)	0.0499 (12)	
C1	0.8474 (5)	0.2552 (2)	0.6297 (5)	0.0398 (12)	
H1A	0.8777	0.2587	0.5581	0.048*	
H1B	0.8444	0.2897	0.6626	0.048*	
C2	0.9392 (5)	0.2243 (2)	0.7210 (5)	0.0379 (12)	
C3	1.0029 (6)	0.1843 (3)	0.6852 (6)	0.0578 (17)	
H3	0.9881	0.1760	0.6033	0.069*	
C4	1.0897 (7)	0.1561 (3)	0.7722 (9)	0.081 (2)	
H4	1.1329	0.1291	0.7485	0.097*	
C5	1.1106 (6)	0.1685 (4)	0.8913 (7)	0.075 (2)	
C6	1.0492 (6)	0.2071 (3)	0.9296 (6)	0.065 (2)	
H6	1.0640	0.2148	1.0118	0.078*	
C7	0.9642 (5)	0.2348 (3)	0.8437 (5)	0.0491 (15)	
H7	0.9219	0.2617	0.8692	0.059*	
C8	0.6182 (5)	0.1582 (2)	0.4614 (5)	0.0432 (13)	
H8A	0.5695	0.1351	0.4969	0.052*	
H8B	0.5676	0.1685	0.3821	0.052*	
C9	0.7277 (5)	0.1289 (2)	0.4448 (5)	0.0434 (13)	
C10	0.7677 (6)	0.1362 (2)	0.3425 (6)	0.0534 (15)	
H10	0.7243	0.1586	0.2824	0.064*	
C11	0.8708 (7)	0.1112 (3)	0.3262 (8)	0.070 (2)	
H11	0.8977	0.1164	0.2568	0.084*	
C12	0.9309 (7)	0.0787 (3)	0.4153 (10)	0.078 (2)	
C13	0.8933 (8)	0.0695 (3)	0.5164 (8)	0.077 (2)	
H13	0.9357	0.0460	0.5743	0.093*	
C14	0.7925 (7)	0.0953 (3)	0.5329 (6)	0.0645 (19)	
H14	0.7676	0.0901	0.6034	0.077*	
C15	0.4941 (5)	0.2654 (2)	0.5752 (5)	0.0413 (13)	
H15A	0.4839	0.2659	0.6569	0.050*	
H15B	0.4962	0.3010	0.5478	0.050*	
C16	0.3905 (5)	0.2365 (2)	0.4919 (5)	0.0384 (13)	
C17	0.3326 (6)	0.1950 (3)	0.5316 (5)	0.0514 (15)	
H17	0.3547	0.1861	0.6134	0.062*	
C18	0.2426 (6)	0.1667 (3)	0.4506 (7)	0.0626 (18)	
H18	0.2041	0.1391	0.4779	0.075*	
C19	0.2107 (6)	0.1796 (3)	0.3309 (6)	0.0590 (17)	
C20	0.2654 (6)	0.2200 (3)	0.2879 (6)	0.0579 (18)	
H20	0.2435	0.2282	0.2057	0.070*	
C21	0.3534 (5)	0.2481 (3)	0.3690 (5)	0.0484 (14)	
H21	0.3898	0.2761	0.3404	0.058*	
C22	0.6819 (5)	0.3121 (2)	0.3390 (5)	0.0356 (12)	
C23	0.7238 (6)	0.3564 (2)	0.4291 (5)	0.0434 (13)	
H23A	0.8099	0.3507	0.4709	0.052*	
H23B	0.6776	0.3542	0.4895	0.052*	
C24	0.5468 (6)	0.4321 (2)	0.3331 (5)	0.0438 (13)	
C25	0.6014 (7)	0.5209 (2)	0.2852 (6)	0.0654 (19)	
H25	0.6822	0.5091	0.3340	0.078*	
C26	0.5775 (10)	0.5729 (3)	0.3367 (7)	0.104 (4)	
H26A	0.5754	0.5686	0.4196	0.157*	
H26B	0.4998	0.5864	0.2899	0.157*	
H26C	0.6422	0.5967	0.3332	0.157*	
C27	0.6113 (10)	0.5271 (3)	0.1567 (7)	0.092 (3)	
H27A	0.6293	0.4940	0.1264	0.138*	
H27B	0.6763	0.5511	0.1557	0.138*	
H27C	0.5348	0.5400	0.1062	0.138*	
C28	0.3806 (7)	0.4941 (3)	0.2433 (6)	0.0628 (18)	
H28	0.3811	0.5291	0.2098	0.075*	
C29	0.3171 (9)	0.4989 (4)	0.3436 (8)	0.106 (3)	
H29A	0.2324	0.5084	0.3096	0.159*	
H29B	0.3574	0.5251	0.4001	0.159*	
H29C	0.3210	0.4663	0.3850	0.159*	
C30	0.3161 (7)	0.4601 (3)	0.1383 (7)	0.078 (2)	
H30A	0.3625	0.4597	0.0788	0.117*	
H30B	0.2350	0.4736	0.1022	0.117*	
H30C	0.3098	0.4255	0.1668	0.117*	

### Atomic displacement parameters (Å^2^)

**Table:**

	*U*^11^	*U*^22^	*U*^33^	*U*^12^	*U*^13^	*U*^23^
Sn1	0.0307 (2)	0.0347 (2)	0.02739 (18)	−0.00011 (16)	0.00355 (13)	−0.00373 (14)
S1	0.0613 (10)	0.0373 (8)	0.0522 (9)	0.0002 (7)	0.0088 (7)	−0.0025 (7)
S2	0.0637 (11)	0.0510 (10)	0.0694 (11)	0.0104 (8)	0.0281 (9)	0.0142 (8)
F1	0.084 (3)	0.184 (6)	0.113 (4)	0.071 (4)	0.003 (3)	0.070 (4)
F2	0.085 (3)	0.071 (3)	0.226 (7)	0.025 (3)	0.063 (4)	−0.010 (4)
F3	0.068 (3)	0.118 (4)	0.085 (3)	−0.044 (3)	0.001 (2)	−0.019 (3)
O1	0.048 (2)	0.036 (2)	0.0333 (19)	0.0021 (17)	0.0058 (16)	0.0027 (16)
O2	0.051 (2)	0.039 (2)	0.0285 (18)	0.0038 (18)	0.0116 (16)	−0.0028 (15)
N1	0.072 (4)	0.038 (3)	0.037 (2)	0.011 (3)	0.010 (2)	0.003 (2)
C1	0.031 (3)	0.050 (3)	0.037 (3)	−0.006 (3)	0.006 (2)	0.004 (2)
C2	0.027 (3)	0.050 (3)	0.034 (3)	−0.002 (2)	0.004 (2)	0.005 (2)
C3	0.056 (4)	0.063 (4)	0.053 (4)	0.008 (3)	0.013 (3)	−0.009 (3)
C4	0.064 (5)	0.068 (5)	0.105 (7)	0.031 (4)	0.012 (4)	0.001 (5)
C5	0.048 (4)	0.102 (6)	0.066 (5)	0.022 (4)	−0.001 (3)	0.034 (4)
C6	0.046 (4)	0.111 (6)	0.035 (3)	0.015 (4)	0.005 (3)	0.014 (4)
C7	0.035 (3)	0.073 (4)	0.036 (3)	0.006 (3)	0.004 (2)	−0.006 (3)
C8	0.043 (3)	0.043 (3)	0.043 (3)	−0.009 (3)	0.009 (2)	−0.014 (2)
C9	0.049 (3)	0.033 (3)	0.045 (3)	−0.007 (3)	0.008 (3)	−0.011 (2)
C10	0.060 (4)	0.040 (3)	0.063 (4)	0.003 (3)	0.021 (3)	0.002 (3)
C11	0.071 (5)	0.050 (4)	0.101 (6)	−0.001 (4)	0.044 (4)	−0.003 (4)
C12	0.051 (4)	0.039 (4)	0.143 (8)	0.010 (3)	0.021 (5)	−0.013 (5)
C13	0.085 (6)	0.041 (4)	0.092 (6)	0.020 (4)	0.001 (5)	0.001 (4)
C14	0.087 (5)	0.041 (4)	0.062 (4)	0.007 (4)	0.013 (4)	−0.001 (3)
C15	0.040 (3)	0.047 (3)	0.036 (3)	0.006 (3)	0.009 (2)	−0.002 (2)
C16	0.030 (3)	0.048 (3)	0.037 (3)	0.012 (2)	0.009 (2)	0.004 (2)
C17	0.049 (4)	0.064 (4)	0.043 (3)	0.003 (3)	0.015 (3)	0.010 (3)
C18	0.053 (4)	0.068 (5)	0.069 (5)	−0.013 (4)	0.019 (3)	0.011 (4)
C19	0.041 (3)	0.070 (5)	0.063 (4)	−0.011 (3)	0.009 (3)	−0.007 (4)
C20	0.040 (3)	0.088 (5)	0.039 (3)	−0.004 (3)	−0.003 (3)	0.003 (3)
C21	0.044 (3)	0.060 (4)	0.037 (3)	−0.008 (3)	0.004 (3)	0.007 (3)
C22	0.032 (3)	0.040 (3)	0.032 (3)	0.007 (2)	0.003 (2)	0.000 (2)
C23	0.052 (3)	0.041 (3)	0.032 (3)	0.007 (3)	0.002 (2)	−0.002 (2)
C24	0.065 (4)	0.036 (3)	0.032 (3)	0.003 (3)	0.016 (3)	−0.003 (2)
C25	0.093 (5)	0.037 (4)	0.056 (4)	−0.002 (4)	0.002 (4)	0.004 (3)
C26	0.179 (10)	0.046 (4)	0.062 (5)	0.012 (5)	−0.013 (6)	−0.010 (4)
C27	0.143 (8)	0.066 (5)	0.076 (5)	−0.024 (5)	0.044 (6)	0.006 (4)
C28	0.079 (5)	0.054 (4)	0.054 (4)	0.021 (4)	0.015 (3)	0.009 (3)
C29	0.133 (8)	0.113 (8)	0.085 (6)	0.074 (7)	0.050 (6)	0.019 (5)
C30	0.073 (5)	0.088 (6)	0.066 (5)	0.010 (4)	0.004 (4)	0.023 (4)

### Geometric parameters (Å, °)

**Table:**

Sn1—C8	2.133 (5)	C12—C13	1.352 (12)
Sn1—C15	2.151 (5)	C13—C14	1.369 (10)
Sn1—C1	2.155 (5)	C13—H13	0.9300
Sn1—O2^i^	2.162 (3)	C14—H14	0.9300
Sn1—O1	2.500 (4)	C15—C16	1.493 (8)
S1—C24	1.782 (6)	C15—H15A	0.9700
S1—C23	1.787 (6)	C15—H15B	0.9700
S2—C24	1.655 (6)	C16—C21	1.387 (8)
F1—C5	1.351 (8)	C16—C17	1.389 (8)
F2—C12	1.391 (8)	C17—C18	1.383 (9)
F3—C19	1.361 (7)	C17—H17	0.9300
O1—C22	1.232 (6)	C18—C19	1.359 (9)
O2—C22	1.265 (6)	C18—H18	0.9300
O2—Sn1^ii^	2.162 (3)	C19—C20	1.366 (9)
N1—C24	1.332 (7)	C20—C21	1.368 (8)
N1—C28	1.474 (8)	C20—H20	0.9300
N1—C25	1.490 (9)	C21—H21	0.9300
C1—C2	1.488 (7)	C22—C23	1.525 (7)
C1—H1A	0.9700	C23—H23A	0.9700
C1—H1B	0.9700	C23—H23B	0.9700
C2—C3	1.378 (8)	C25—C27	1.510 (10)
C2—C7	1.382 (8)	C25—C26	1.516 (10)
C3—C4	1.396 (10)	C25—H25	0.9800
C3—H3	0.9300	C26—H26A	0.9600
C4—C5	1.356 (11)	C26—H26B	0.9600
C4—H4	0.9300	C26—H26C	0.9600
C5—C6	1.349 (11)	C27—H27A	0.9600
C6—C7	1.373 (9)	C27—H27B	0.9600
C6—H6	0.9300	C27—H27C	0.9600
C7—H7	0.9300	C28—C30	1.506 (10)
C8—C9	1.500 (8)	C28—C29	1.508 (10)
C8—H8A	0.9700	C28—H28	0.9800
C8—H8B	0.9700	C29—H29A	0.9600
C9—C10	1.372 (8)	C29—H29B	0.9600
C9—C14	1.380 (9)	C29—H29C	0.9600
C10—C11	1.382 (9)	C30—H30A	0.9600
C10—H10	0.9300	C30—H30B	0.9600
C11—C12	1.351 (11)	C30—H30C	0.9600
C11—H11	0.9300		
			
C8—Sn1—C15	109.5 (2)	Sn1—C15—H15B	110.3
C8—Sn1—C1	121.1 (2)	H15A—C15—H15B	108.6
C15—Sn1—C1	127.8 (2)	C21—C16—C17	117.0 (5)
C8—Sn1—O2^i^	88.72 (19)	C21—C16—C15	120.8 (5)
C15—Sn1—O2^i^	98.59 (18)	C17—C16—C15	122.1 (5)
C1—Sn1—O2^i^	94.94 (17)	C18—C17—C16	120.7 (6)
C8—Sn1—O1	83.41 (18)	C18—C17—H17	119.6
C15—Sn1—O1	90.48 (18)	C16—C17—H17	119.6
C1—Sn1—O1	83.39 (17)	C19—C18—C17	119.7 (6)
O2^i^—Sn1—O1	169.58 (13)	C19—C18—H18	120.2
C24—S1—C23	103.0 (3)	C17—C18—H18	120.2
C22—O1—Sn1	139.2 (3)	C18—C19—F3	120.0 (6)
C22—O2—Sn1^ii^	131.5 (3)	C18—C19—C20	121.6 (6)
C24—N1—C28	123.0 (6)	F3—C19—C20	118.4 (6)
C24—N1—C25	121.9 (6)	C21—C20—C19	118.3 (6)
C28—N1—C25	114.9 (5)	C21—C20—H20	120.9
C2—C1—Sn1	117.4 (4)	C19—C20—H20	120.9
C2—C1—H1A	107.9	C20—C21—C16	122.7 (6)
Sn1—C1—H1A	107.9	C20—C21—H21	118.6
C2—C1—H1B	107.9	C16—C21—H21	118.6
Sn1—C1—H1B	107.9	O1—C22—O2	125.6 (5)
H1A—C1—H1B	107.2	O1—C22—C23	119.4 (5)
C3—C2—C7	117.8 (5)	O2—C22—C23	114.8 (5)
C3—C2—C1	120.7 (5)	C22—C23—S1	117.6 (4)
C7—C2—C1	121.5 (5)	C22—C23—H23A	107.9
C2—C3—C4	119.8 (6)	S1—C23—H23A	107.9
C2—C3—H3	120.1	C22—C23—H23B	107.9
C4—C3—H3	120.1	S1—C23—H23B	107.9
C5—C4—C3	119.6 (7)	H23A—C23—H23B	107.2
C5—C4—H4	120.2	N1—C24—S2	125.6 (5)
C3—C4—H4	120.2	N1—C24—S1	115.1 (5)
C4—C5—F1	118.0 (8)	S2—C24—S1	119.3 (3)
C4—C5—C6	122.1 (6)	N1—C25—C27	111.3 (6)
F1—C5—C6	119.9 (7)	N1—C25—C26	114.3 (7)
C5—C6—C7	118.1 (6)	C27—C25—C26	110.5 (6)
C5—C6—H6	120.9	N1—C25—H25	106.7
C7—C6—H6	120.9	C27—C25—H25	106.7
C6—C7—C2	122.5 (6)	C26—C25—H25	106.7
C6—C7—H7	118.8	C25—C26—H26A	109.5
C2—C7—H7	118.8	C25—C26—H26B	109.5
C9—C8—Sn1	114.9 (4)	H26A—C26—H26B	109.5
C9—C8—H8A	108.5	C25—C26—H26C	109.5
Sn1—C8—H8A	108.5	H26A—C26—H26C	109.5
C9—C8—H8B	108.5	H26B—C26—H26C	109.5
Sn1—C8—H8B	108.5	C25—C27—H27A	109.5
H8A—C8—H8B	107.5	C25—C27—H27B	109.5
C10—C9—C14	118.6 (6)	H27A—C27—H27B	109.5
C10—C9—C8	120.3 (6)	C25—C27—H27C	109.5
C14—C9—C8	121.1 (6)	H27A—C27—H27C	109.5
C9—C10—C11	121.9 (7)	H27B—C27—H27C	109.5
C9—C10—H10	119.1	N1—C28—C30	112.4 (6)
C11—C10—H10	119.1	N1—C28—C29	110.5 (6)
C12—C11—C10	117.2 (7)	C30—C28—C29	115.4 (8)
C12—C11—H11	121.4	N1—C28—H28	105.9
C10—C11—H11	121.4	C30—C28—H28	105.9
C11—C12—C13	123.0 (7)	C29—C28—H28	105.9
C11—C12—F2	117.5 (9)	C28—C29—H29A	109.5
C13—C12—F2	119.5 (8)	C28—C29—H29B	109.5
C12—C13—C14	119.4 (7)	H29A—C29—H29B	109.5
C12—C13—H13	120.3	C28—C29—H29C	109.5
C14—C13—H13	120.3	H29A—C29—H29C	109.5
C13—C14—C9	120.0 (7)	H29B—C29—H29C	109.5
C13—C14—H14	120.0	C28—C30—H30A	109.5
C9—C14—H14	120.0	C28—C30—H30B	109.5
C16—C15—Sn1	107.0 (3)	H30A—C30—H30B	109.5
C16—C15—H15A	110.3	C28—C30—H30C	109.5
Sn1—C15—H15A	110.3	H30A—C30—H30C	109.5
C16—C15—H15B	110.3	H30B—C30—H30C	109.5
			
C8—Sn1—O1—C22	−176.6 (5)	C1—Sn1—C15—C16	162.0 (3)
C15—Sn1—O1—C22	73.8 (5)	O2^i^—Sn1—C15—C16	−94.8 (4)
C1—Sn1—O1—C22	−54.2 (5)	O1—Sn1—C15—C16	80.0 (4)
O2^i^—Sn1—O1—C22	−135.5 (7)	Sn1—C15—C16—C21	−87.7 (6)
C8—Sn1—C1—C2	−70.7 (5)	Sn1—C15—C16—C17	88.0 (6)
C15—Sn1—C1—C2	125.7 (4)	C21—C16—C17—C18	0.2 (9)
O2^i^—Sn1—C1—C2	20.8 (4)	C15—C16—C17—C18	−175.6 (6)
O1—Sn1—C1—C2	−148.9 (4)	C16—C17—C18—C19	0.3 (10)
Sn1—C1—C2—C3	86.3 (6)	C17—C18—C19—F3	−180.0 (6)
Sn1—C1—C2—C7	−94.2 (6)	C17—C18—C19—C20	−0.1 (11)
C7—C2—C3—C4	−0.3 (10)	C18—C19—C20—C21	−0.6 (11)
C1—C2—C3—C4	179.2 (6)	F3—C19—C20—C21	179.3 (6)
C2—C3—C4—C5	0.0 (12)	C19—C20—C21—C16	1.2 (10)
C3—C4—C5—F1	179.7 (7)	C17—C16—C21—C20	−1.0 (9)
C3—C4—C5—C6	0.5 (13)	C15—C16—C21—C20	174.9 (6)
C4—C5—C6—C7	−0.8 (13)	Sn1—O1—C22—O2	177.0 (3)
F1—C5—C6—C7	−179.9 (7)	Sn1—O1—C22—C23	1.7 (8)
C5—C6—C7—C2	0.5 (11)	Sn1^ii^—O2—C22—O1	−8.5 (8)
C3—C2—C7—C6	0.0 (9)	Sn1^ii^—O2—C22—C23	166.9 (3)
C1—C2—C7—C6	−179.5 (6)	O1—C22—C23—S1	−162.0 (4)
C15—Sn1—C8—C9	176.0 (4)	O2—C22—C23—S1	22.3 (6)
C1—Sn1—C8—C9	9.6 (5)	C24—S1—C23—C22	72.4 (5)
O2^i^—Sn1—C8—C9	−85.4 (4)	C28—N1—C24—S2	−9.3 (8)
O1—Sn1—C8—C9	87.8 (4)	C25—N1—C24—S2	176.7 (4)
Sn1—C8—C9—C10	−97.9 (6)	C28—N1—C24—S1	171.9 (4)
Sn1—C8—C9—C14	80.2 (6)	C25—N1—C24—S1	−2.1 (7)
C14—C9—C10—C11	−0.4 (10)	C23—S1—C24—N1	176.2 (4)
C8—C9—C10—C11	177.7 (6)	C23—S1—C24—S2	−2.6 (4)
C9—C10—C11—C12	0.3 (11)	C24—N1—C25—C27	100.4 (7)
C10—C11—C12—C13	1.0 (12)	C28—N1—C25—C27	−74.0 (8)
C10—C11—C12—F2	180.0 (6)	C24—N1—C25—C26	−133.4 (6)
C11—C12—C13—C14	−2.2 (13)	C28—N1—C25—C26	52.1 (7)
F2—C12—C13—C14	178.8 (7)	C24—N1—C28—C30	−56.2 (8)
C12—C13—C14—C9	2.1 (12)	C25—N1—C28—C30	118.2 (7)
C10—C9—C14—C13	−0.8 (10)	C24—N1—C28—C29	74.3 (8)
C8—C9—C14—C13	−178.9 (6)	C25—N1—C28—C29	−111.4 (7)
C8—Sn1—C15—C16	−3.1 (4)		

Symmetry codes: (i) *x*, −*y*+1/2, *z*+1/2; (ii) *x*, −*y*+1/2, *z*−1/2.
